# Potential intravenous drug incompatibilities in a pediatric unit

**DOI:** 10.1590/S1679-45082016AO3723

**Published:** 2016

**Authors:** Karla Dalliane Batista Leal, Ramon Weyler Duarte Leopoldino, Rand Randall Martins, Lourena Mafra Veríssimo

**Affiliations:** 1 Universidade Federal do Rio Grande do Norte, Natal, RN, Brazil.

**Keywords:** Risk factors, Drug therapy, Administration, intravenous, Drug incompatibility, Child

## Abstract

**Objective:**

To investigate potential intravenous drug incompatibilities and related risk factors in a pediatric unit.

**Methods:**

A cross-sectional analytical study conducted in the pediatric unit of a university hospital in Brazil. Data on prescriptions given to children aged 0-15 years from June to October 2014 were collected. Prescriptions that did not include intravenous drugs and prescriptions with incomplete dosage regimen or written in poor handwriting were excluded. Associations between variables and the risk of potential incompatibility were investigated using the Student’s *t* test and ANOVA; the level of significance was set at 5% (p<0.05). Relative risks were calculated for each drug involved in potential incompatibility with 95% confidence interval.

**Results:**

A total of 222 children participated in the study; 132 (59.5%) children were male and 118 (53.2%) were aged between 0 and 2 years. The mean length of stay was 7.7±2.3 days. Dipyrone, penicillin G and ceftriaxona were the most commonly prescribed drugs. At least one potential incompatibility was detected in about 85% of children (1.2 incompatibility/patient ratio). Most incompatibilities detected fell into the non-tested (93.4%), precipitation (5.5%), turbidity (0.7%) or chemical decomposition (0.4%) categories. The number of drugs and prescription of diazepam, phenytoin, phenobarbital or metronidazole were risk factors for potential incompatibility.

**Conclusion:**

Most pediatric prescriptions involved potential incompatibilities, with higher prevalence of non-tested incompatibilities. The number of drugs and prescription of diazepam, phenobarbital, phenytoin or metronidazole were risk factors for potential incompatibilities.

## RESUMO

**Objetivo:**

Avaliar o potencial de incompatibilidade dos medicamentos intravenosos, identificando possíveis fatores de risco em uma unidade pediátrica.

**Métodos:**

Trata-se de um estudo observacional analítico do tipo transversal realizado na unidade de pediatria de um hospital de ensino no Brasil. Os dados foram coletados de junho a outubro de 2014 a partir da análise das prescrições de crianças (0 a 15 anos) hospitalizadas. Foram excluídas prescrições sem medicamento intravenoso e com posologia incompletas ou grafia inadequada. A associação entre as variáveis e o risco de potenciais incompatibilidades foi determinada pelo teste *t* de Student e ANOVA, considerando significativo para p<0,05. Calculou-se o risco relativo com intervalo de confiança de 95% de cada medicamento envolvido.

**Resultados:**

Duzentos e vinte e duas crianças participaram do estudo, 132 (59,5%) eram do gênero masculino, 118 (53,2%) tinham idade entre 0 a 2 anos e estiveram internados em média 7,7±2,3 dias. Os medicamentos mais prescritos foram dipirona, penicilina G e ceftriaxona. Quase 85% das crianças apresentaram ao menos uma potencial incompatibilidade, razão de 1,2 incompatibilidades/paciente. Os tipos de incompatibilidades mais comuns foram: não testada (93,4%), precipitação (5,5%), turbidez (0,7%) e decomposição química (0,4%). Os fatores associados a potenciais incompatibilidades foram: número de medicamentos e a prescrição dos medicamentos diazepam, fenitoína, fenobarbital e metronidazol.

**Conclusão:**

A maioria das prescrições pediátricas apresentou potenciais incompatibilidades e a incompatibilidade não testada foi o tipo mais comum. O número de medicamentos e a prescrição dos medicamentos diazepam, fenobarbital, fenitoína e metronidazol foram fatores de risco para potenciais incompatibilidades.

## INTRODUCTION

The clinical condition of hospitalized patients often requires intravenous drug therapy, with potential exposure of patients to several risks and harms.^(1,2)^ The complexity of intravenous drug therapy, drug administration in particular, is the major cause of such problems. Intravenous medication administration is a complex procedure involving several steps, and is therefore prone to error.^(3,4)^

Medication administration errors are common in pediatric medicine and chances of occurrence around 70% have been reported.^(4)^ Children are more vulnerable to medication administration errors due to off-label drug use, drug dose variation, lack of standardized dosage regimen, dose calculation based on body weight, immaturity of body organs and systems and inability to communicate effectively.^(3,5)^ In a study by Vijayakumar et al.^(2)^ incompatibility issues accounted for most problems related to intravenous drug administration.

Incompatibility refers to unexpected physical and/or chemical interactions between two or more substances in a mixture, with therapeutic safety and efficacy compromise.^(6)^ Incompatibilities may be physical or chemical in nature. Physical incompatibilities result in visible reactions (*e.g.*, precipitation, turbidity, viscosity, color changes and gas production) while chemical incompatibilities are related to drug degradation due to hydrolysis, oxidation or covalent chemical reactions.^(6,7)^ Several factors have been associated with the occurrence of drug incompatibilities, potential of hydrogen (pH) being the most significant. Most drugs are either acids or weak bases; therefore, minimal changes in pH can translate into incompatibility.^(6,8)^

The incompatibilities may have several consequences, from simple catheter obstruction to patient death. Physical incompatibilities are more likely to occur in clinical practice.^(8)^ Therefore, the multidisciplinary teams must be aware of the problem, bearing in mind the paucity of literature data, lack of knowledge and/or limited health professional training. Pharmacists receive drug-centered education and are better qualified to advise on drug incompatibility issues. Therefore, these professionals should be part of multidisciplinary healthcare teams.^(2,7,9,10)^

## OBJECTIVE

To investigate potential intravenous drug incompatibilities and related risk factors in a pediatric unit.

## METHODS

### Study design and population

A cross-sectional analytical study carried out at the pediatric unit of a medium-sized public university hospital, located in Santa Cruz (RN), Brazil. Prescriptions given to children aged 15 years or under, with a minimum of 24-hour inpatient hospital stay and receiving at least one intravenous medication were included in the sample. Prescriptions containing incomplete dosage regimens or written in poor handwriting were excluded. Prescriptions given from June to October 2014 were selected out of convenience.

### Data collection

Data collection was based on prescription analysis. The following variables were considered: gender, age, cause of hospitalization, number of intravenous medications, dosage, length of inpatient hospital stay and presence and type of potential incompatibilities (PI). Medications were grouped according to the World Health Organization (WHO) Anatomical Therapeutic Chemical Classification System (ATC).^(11)^

### Potential incompatibilities

Prescription of one or more intravenous drugs reported to be physically and chemically incompatible were defined as potential incompatibility (PI) in this study. Incompatibilities could not be actually confirmed; therefore the analysis was based on PIs detected in prescriptions. Potential incompatibility classification was based on Micromedex^®^ version 2.0.^(12)^ The following categories were considered: precipitation, turbidity, chemical decomposition, color changes, variable and non-tested. Variable and non-tested categories were included in the PI classification list due to the paucity of studies or conflicting literature data on the drug combinations involved; incompatibility risks in these cases were assumed to be similar to other PIs.

### Sample size and statistical analysis

Sample size calculation was based on the following assumptions: 50% PI prevalence, 7% absolute precision plus 10% to account for potential losses. The level of significance was set at 5%. Data extracted from 222 prescriptions and arranged in Excel spreadsheets (software Excel^®^ 2010) were analyzed using the Statistical Package for the Social Sciences SPSS^®^ version 18.0. Variables were expressed as mean ± standard deviation and absolute or relative frequency, as needed. Drug/patient and PI/patient ratios were calculated by dividing the number of drugs or PIs by the total number of patients, respectively. Associations between variables and the risk of PI were investigated using the Student’s *t* test and ANOVA. Significance was indicated by p<0.05. Whenever PIs were detected, relative risks were calculated for each drug with 95% confidence interval.

This study was approved by the Research Ethics Committee of the *Faculdade de Ciências e Saúde do Trairi*, *Universidade Federal do Rio Grande*, under CAAE number 30951614.1.0000.5568.

## RESULTS

A total of 222 children were included in the study; in that, 132 (59.5%) were male and 118 were aged between 0 and 2 years (53.2%). The mean length of stay was 7.7±2.3 days. Respiratory diseases were the major cause of hospitalization. Drug/patient ratio corresponded to 2.5, with 135 (60.8%) children receiving up to two drugs. Analgesics (dipyrone), penicillins (penicillin potassium) and other beta-lactams (ceftriaxona) were the most commonly prescribed drugs (Table 1).

**Table 1:** Sample characterization

Demographic and clinical characteristics	n (%)
Male gender	132 (59.5)
Age	
0 to 2 years (n, %)	118 (53.2)
2.1 to 15 years (n, %)	104 (46.8)
Cause of hospitalization	
Respiratory diseases	104 (46.8)
AGEC^a^	51 (23.0)
Cellulitis	16 (7.2)
Infections	23 (10.4)
Other	28 (12.6)
Drugs	
Up to 2	135 (60.8)
3 or more	87 (39.2)
Most commonly prescribed drug classes (ATC/WHO)	
Analgesics e antipyretics (N02B)	176 (32.2)
Penicillins (J01C)	127 (23.2)
Other beta-lactams (J01D)	57 (10.4)
Systemic corticosteroids (H02A)	49 (9.0)
Aminoglycosides (J01G)	38 (6.9)
Antiemetic agents (A04A)	28 (5.1)
Prokinetic agents (A03F)	27 (4.9)
Gastro-protective agents (A02B)	21 (3.8)
Anticonvulsants (N03A)	5 (0.9)
Anxiolytics (N05B)	4 (0.7)

AGEC^a^: acute gastroenterocolitis. ATC: Anatomical Therapeutic Chemical Classification System; WHO: World Health Organization.

Potential intravenous drug incompatibilities were detected in 185 (83.3%) children, with 1.2 PI/patient ratio (Table 2). Most incompatibilities detected in prescriptions fell into the non-tested, precipitation, turbidity or chemical decomposition categories (93.4%, 5.5%, 0.7% and 0.4%, respectively).

**Table 2:** Potential intravenous drug incompatibilities in pediatric prescriptions and respective types

	n (%)
Prescriptions	
With PI	185 (83.3)
Without PI	37 (16.7)
Type of incompatibility	
Precipitation	15 (5.5)
Turbidity	2 (0.7)
Decomposition	1 (0.4)
Non-tested	255 (93.4)

PI: potential incompatibility.

The number of drugs prescribed was a risk factor for PI (Table 3). Prescription of diazepam, phenytoin, phenobarbital or metronidazole was also associated with increased PI risks (Table 4).

**Table 3:** Risk factors for potential intravenous drug incompatibility

Demographic and clinical characteristics	PI		

	M	SD	p value
Gender			
Male	1.4	0.7	0.583^b^
Female	1.5	0.7	
Age			
0 to 2 years	1.4	0.6	0.101^b^
2.1 to 15 years	1.5	0.8	
Hospital stay			
1 to 8 days	1.5	0.8	0.401^b^
9 days or over	1.4	0.7	
Drugs			
Up to 2	1.1	0.2	0.000^b^*
3 or more	2.1	0.7	
Cause of hospitalization			
Respiratory diseases	0.9	0.6	0.623^c^
AGEC^a^	1.1	0.7	
Cellulitis	1.2	0.4	
Infections	1.4	0.5	
Other	0.9	0.5	

*p<0.05.

^b^ Student´s *t* test; ^c^Analysis of variance.

PI: potential incompatibility; M: mean; SD: standard deviation; AGEC^a^: acute gastroenterocolitis.

**Table 4:** Drugs prescribed and risk of potential intravenous drug incompatibility

Drugs	Prescriptions	PI	RR	95%CI	
	n (%)	n (%)			
Amikacin	36 (13.2)	4 (11.1)	1.77	0.59	5.29
Acyclovir	1 (0.4)	1 (2.8)	15.60	0.96	254.71
Ceftriaxone	44 (16.1)	3 (8.3)	1.04	0.31	3.53
Diazepam	4 (1.5)	8 (22.2)	38.79*	11.01	136.59
Epinephrine	2 (0.7)	1 (2.8)	7.79	0.69	87.97
Phenytoin	2 (0.7)	3 (8.3)	24.77*	4.00	153.41
Phenobarbital	3 (1.1)	5 (13.9)	29.25*	6.68	128.02
Furosemide	4 (1.5)	1 (2.8)	3.88	0.42	35.63
Metoclopramide	27 (9.9)	1 (2.8)	0.55	0.07	4.17
Metronidazole	4 (1.5)	2 (5.6)	7.99*	1.41	45.15
Ondansetron	21 (7.7)	3 (8.3)	2.28	0.65	8.03
Oxacillin	22 (8.1)	1 (2.8)	0.68	0.09	5.21
Penicillin potassium	103 (37.7)	3 (8.3)	0.39	0.12	1.30

* Significant relative risk.

PI: potencial incompatibility; RR: relative risk; 95%CI: 95% confidence interval.

## DISCUSSION

This study suggests PIs are common in pediatric medicine and emphasizes the significance of intravenous drug administration safety. Studies investigating incompatibilities in pediatric medicine are scarce; in fact, this is the first Brazilian study on the topic and the first to include incompatibility risk assessment.

Different from trials reporting low numbers of incompatible drug combinations in intensive care settings,^(1,9,13)^ approximately 80% of children in this study were exposed to PIs. This reflects the effect of preventive measures and strategies in hospitals; however, incompatibilities may still interfere with medication administration.^(2)^

Studies investigating drug compatibility are scarce and tend to be ill-suited for clinical application.^(8)^ High prevalence of non-tested incompatibilities was documented in this study, dipyrone being the major drug involved. Dipyrone is a classical example of non-tested PI: the fact that the drug is not on the market in many countries, including the USA, translates into lack of interest in related publications by countries with more robust scientific tradition.^(14)^ The abundance of novel drugs with common off-label pediatric use in the market also contributes to this scenario. To illustrate the point, chemical decomposition of a potassium penicillin-phenobarbital combination given at 500,000IU/ml and 65mg/ml, respectively, has been reported; however, lower concentrations, such as those used in pediatric medicine have not been tested.^(12)^ Lack of data on drug compatibility promotes unsafe drug use in light of the risk of prescribing potentially incompatible drug combinations, not to mention the risk of infections and thrombosis posed by unnecessary venous cannulation.^(8)^

These incompatibilities are strongly associated with the number of drugs prescribed. Similar to drug interactions, associations between the number of drugs prescribed and PI have been reported.^(15)^ The inclusion of drugs like diazepam, phenobarbital, phenytoin and metronidazole in prescriptions is also known to be a risk factor for drug incompatibility. The association of these drugs with PI may reflect reported incompatibilities with dipyrone and penicillin potassium, the most commonly prescribed drugs.^(12)^ Also, phenobarbital and phenytoin are drugs with extreme pH; given pH is the major factor behind drug incompatibility, such products are more likely to exhibit chemical and physico-chemical interactions.^(6,8)^

Drug administration is a complex procedure and caution is required to avoid medication administration errors and/or incompatibility issues.^(4)^ The following preventive measures are recommended: nursing team guidance and training in drug preparation, administration and potential incompatibilities; implementation of standard operating procedures and distribution of flyers containing data on the most commonly used drugs in clinical practice and instructions for multi-lumen catheter manipulation.^(1,13,16,17)^ Pharmacists are qualified to instruct multidisciplinary teams in drug related problem-solving scenarios; therefore, the involvement of these professionals in drug therapy decisions translates into significant benefits to healthcare services.^(2)^

As previously mentioned, this is the first study on intravenous drug incompatibilities in pediatric medicine, with the added contribution of PI risk factor analysis. The major limitation of this study is the restriction of the analysis to prescriptions, with no data on medication administration procedures as regards PI prevention. Specific focus on prescription and PI and the exclusion of solutions, electrolytes and parenteral nutrition from the analysis are additional limitations; also, cross-sectional experimental design precluded detailed investigation of patient-specific incompatibilities.

Further studies are required for improved understanding of intravenous drug incompatibility issues and ensuing consequences in clinical practice. Future work aimed at incompatibility risk assessment via cross-sectional studies and studies involving other medical centers are among the purposes of this research group. However, development of novel laboratory assays to investigate potential physical and chemical incompatibilities in non-tested drug combinations is also crucial.

## CONCLUSION

Potential incompatibilities were detected in most pediatric prescriptions in this study, with higher prevalence of non-tested incompatibilities. The number of drugs prescribed and the prescription of diazepam, phenobarbital, phenytoin or metronidazole were associated with higher PI risks.
